# Efficacy of corticosteroids in patients with acute respiratory distress syndrome: a meta-analysis

**DOI:** 10.1080/07853890.2024.2381086

**Published:** 2024-08-21

**Authors:** Guowei Li, Dunfan Chen, Feng Gao, Wei Huang, Jin Wang, Yonglin Li, Baijian Chen, Yuejia Zhong, Rui Chen, Manhua Huang

**Affiliations:** Emergency Department, The Second Affiliated Hospital of Guangzhou University of Chinese Medicine, Guangzhou, China

**Keywords:** Acute respiratory distress syndrome, corticosteroid, meta-analysis

## Abstract

**Background:**

Acute respiratory distress syndrome (ARDS), are respiratory diseases with high morbidity and mortality. Clinical trials investigating the efficacy of corticosteroids in the treatment of ARDS often yield contradictory results. We hereby conducted a systematic review and meta-analysis to investigate the efficacy of corticosteroids in ARDS management.

**Materials and Methods:**

We conducted a search for randomized clinical trials (RCT) and observational studies that utilized corticosteroids for patients with ARDS in Web of Science, PubMed, and Embase. The primary outcome was mortality. Risk of bias was assessed using Cochrane or NOS scales. Statistical effect size was analyzed using the Mantel-Haenszel method.

**Results:**

A total of 20 studies, comprising 11 observational studies and 9 RCTs, were eligible for analysis. In RCTs, corticosteroids were associated with a reduction of mortality in ARDS patients (relative risk [RR] = 0.80, 95%CI: 0.71-0.91, *p* = 0.001). Further subgroup analysis indicated that specific variables, such as low-dose (RR = 0.81; 95%CI: 0.67-0.98; *p* = 0.034), methylprednisolone (RR = 0.70; 95%CI: 0.49-0.98; *p* = 0.035), and dexamethasone (RR = 0.82; 95%CI: 0.69-0.98; *p* = 0.029) were associated with mortality among patients receiving corticosteroids. However, in observational studies, corticosteroids increased the risk of death (RR = 1.16, 95%CI: 1.04-1.29; *p* = 0.001). Subgroup analysis showed that the use of high-dose corticosteroids was associated with higher patient mortality (RR = 1.20; 95%CI: 1.04-1.38; *p* = 0.001).

**Conclusions:**

The efficacy of corticosteroids on the mortality of ARDS differed by the type and dosage of corticosteroids used, as well as the etiologies. Current data do not support routine use of corticosteroids in ARDS since protective effects were observed in RCTs but increased mortality was found in observational studies. More well designed and large clinical trials are needed to specify the favorable subgroups for corticosteroid therapy.

## KEY MESSAGES

Corticosteroid use may reduce the risk of death in patients with acute respiratory distress syndrome (ARDS) according to randomized controlled trials.

Observational studies indicate that corticosteroid use may increase the risk of death in non-COVID-19 ARDS patients but not in COVID-19 ARDS patients.

Both regular and low-dose corticosteroids show benefits in reducing mortality in RCTs, but observational studies associate these doses with increased mortality.

## Introduction

Acute respiratory distress syndrome (ARDS) is a respiratory disease characterized by diffuse alveolar damage, hypoxemia, and poor lung compliance as a result of a severe inflammatory process [1]. Due to the heterogeneity of ARDS regarding to the causes, manifestations, and response to treatment, clinicians are challenged to provide impeccable supportive care. Therefore, ARDS remain associated with high morbidity and mortality. In intensive care units (ICUs) in Shanghai, ARDS occurs in 2% of patients older than 15 years with a 70% mortality rate [2]. According to a study conduceted across 459 ICUs in 50 countries, the period prevalence of ARDS was 10.4% of ICU admissions. Hospital mortality was 34.9% for those with mild, 40.3% for those with moderate, and 46.1% for those with severe ARDS [3]. Furthermore, as a result of the COVID-19 pandemic, many clinical settings have become overburdened with patients with severe ARDS. Early reports suggested that COVID-19-associated ARDS possesses distinct features compared with the classic ones [4]. Exploring effective treatments for ARDS is urgent and significant.

The use of corticosteroids was considered as one of the potential treatments for ARDS by reducing systemic inflammatory response and accelerating the recovery of pulmonary infection. However, the results of clinical trials are often contradictory. In a random clinical trial (RCT) comparing dexamethasone with standard treatment for patients with moderate to severe ARDS, patients treated with dexamethasone had 4.8 more ventilator-free days and a 15% absolute risk reduction in 60-day mortality (21% *vs* 36%) than those who received standard care [5]. In another RCT, early administration of corticosteroids to patients with ARDS appeared promising, with significant reductions in mechanical ventilation, ICU stay, mortality, and infectious complications after receiving low-dose methylprednisolone [6]. However, the use of corticosteroids in patients with persistent ARDS did not yield encouraging results in a multi-center RCT. In the methylprednisolone-treated groups, 60- and 180-day mortality rates were similar, but after 14 days of ARDS onset, 60- and 180-day mortality rates were elevated [7]. Consequently, it is still unclear whether corticosteroids are effective for patients with ARDS.

In this study, a systematic review and meta-analysis was performed to determine whether corticosteroid administration is effective in patients with ARDS. Subgroup analysis regarding dose, type of corticosteroids, and cause of disease was also conducted.

## Materials and methods

This study followed the PRISMA statement [8]. The protocol was registered on PROSPERO with reference number CRD42022381280.

### Search strategy

Until November 8, 2022, we searched Web of Science, PubMed, and Embase to identify randomized clinical trials (RCT) and observational studies pertaining to the use of corticosteroids in patients diagnosed with ARDS. Acute respiratory distress syndrome, ARDS, acute lung injury, corticosteroids, hydrocortisone, methylprednisolone, dexamethasone, randomized controlled trials, and clinical trials were all searched separately or in combination, with English as the only permitted language and only human studies were included. The full search strategy is detailed in the Table S1.

### Selection criteria

Eligible studies were identified and analyzed based on specific criteria related to subjects, interventions, controls, outcomes, and study design. Inclusion criteria encompassed patients diagnosed with ARDS. The intervention group received any corticosteroid treatment. Studies without control group were not considered. The titles and abstracts of the included articles were reviewed by two independent investigators. Any differences between the two investigators were settled through discussion or by a third investigator.

### Data collection

The primary outcome was short-term mortality. Two reviewers independently reviewed all included studies and extracted the following information from each study: authors, year of publication, region of the dataset, COVID-19 status of the patients, study design, corticosteroid dosage, total number of participants and number of events in the treatment group, and total number of participants and number of events in the control group. Any discrepancies were resolved through discussion or at the discretion of a third investigator.

A subgroup analysis was conducted to assess the potential associations between corticosteroids dosage, type of coricosteroids, and COVID-19 status with mortality in patients with ARDS. The classification of corticosteroid doses (low/regular/high dose) was based on the original literature classification.

### Risk of bias assessment

We used a modified version of the Cochrane risk of bias tool to assess risk of bias in RCTs, and a revised version of the Newcastle–Ottawa Scale (NOS) for observational studies. Two reviewers independently assessed risk of bias, resolving disagreements with a third reviewer if necessary.

### Statistical analysis

The Q statistic and the *I*^2^ statistic had test levels of 10% and 50%, respectively. A fixed-effects model was employed for the analysis if *I*^2^ < 50% or *p* > 0.1. A random-effects model was applied otherwise. Following Mantel-Haenszel analysis of dichotomous data, the pooled odds ratio (OR) and matching 95% confidence interval (CI) were computed. Statistics were deemed significant at *p* < 0.05. Stata 15.1 was used to perform all statistical analyses (College Station, TX, USA).

## Results

### Study selection

**Figure 1.**
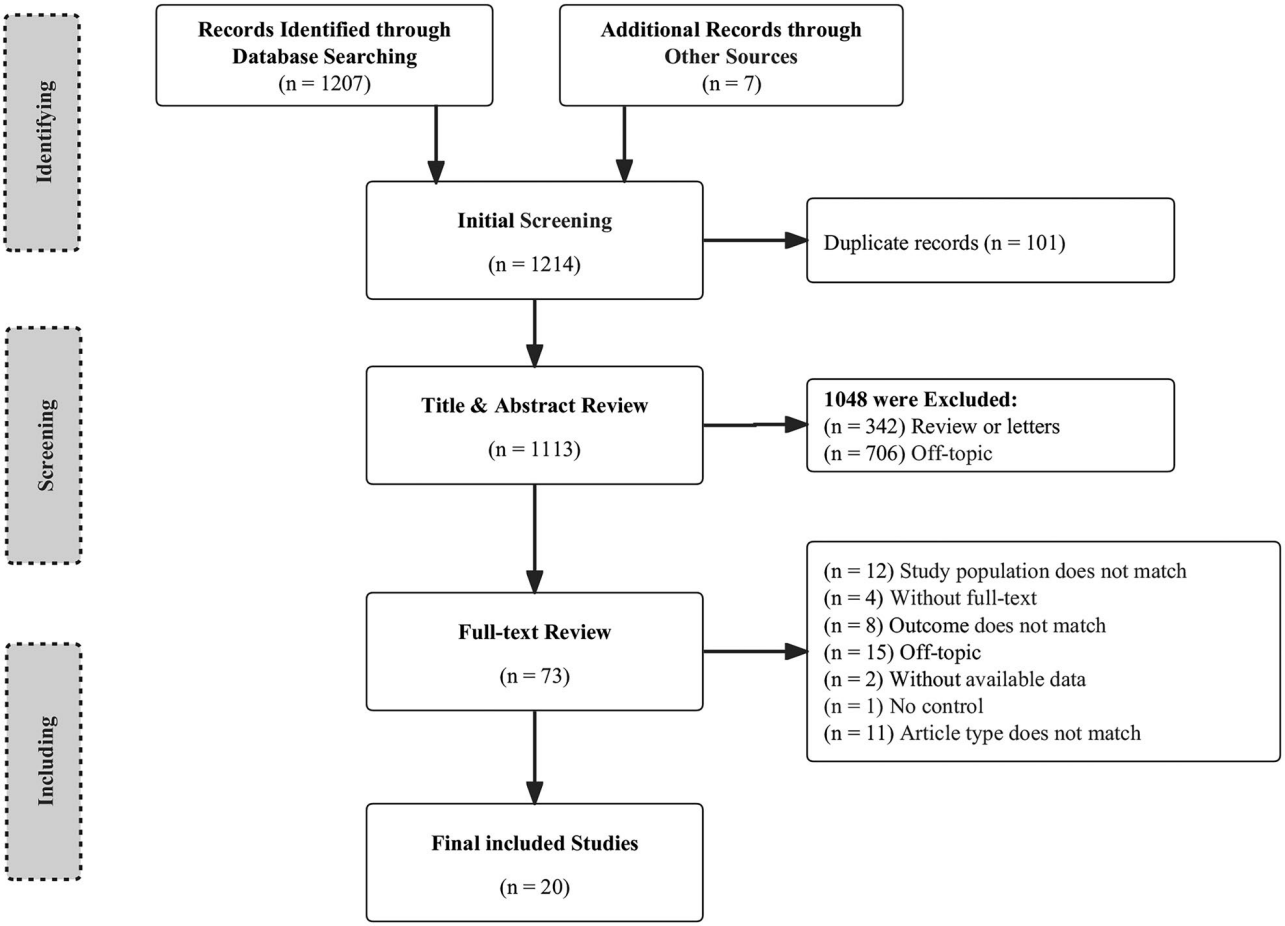
Selection process for articles included in the analysis.

After conducting a comprehensive literature search, a total of 1,214 records were initially identified. Following the removal of duplicates and a careful examination of titles and abstracts, 73 studies remained eligible for further analysis. Subsequently, the full texts of these studies were thoroughly reviewed, leading to the inclusion of 20 papers that met all the predefined inclusion criteria and were consequently utilized in the final analysis (Figure 1).

### Characteristics of the included studies

This meta-analysis included a total of 20 studies involving 3,890 subjects (Table 1). These studies were published between 1998 and 2022, comprising nine RCTs [5–7,17,19–21,23,24] and 11 observational studies [9–16,18,22,25]. Four studies [10,11,16,19] specifically included patients diagnosed with ARDS and concomitant COVID-19, while the other 16 studies included patients diagnosed with ARDS. Eleven studies [5–7,10,12,15,18,19,21,24,25] were conducted in multi centers, while nine studies [9,11,13,14,16,17,20,22,23] were conducted in a single center. Regarding the corticosteroids used in the studies, methylprednisolone was utilized in eight studies [6,7,14,16,20,22,24,25], hydrocortisone in three studies [17,21,23], dexamethasone in two studies [5,19], and multiple kinds of corticosteroids in seven study [9–13,15,18].

**Table 1.** The characteristics of the included studies.

Author	Year	Region	Study design	Subjects	EGC*	Treatment	TE*	EE*	TC*	EC*	Intervention	Covid-19 or not
**RCT**												
Villar [9]	2020	Spain	Multicenter	ARDS	CS	20 mg once daily, Day1-5; 10 mg once daily, Day 6-10	139	33	138	50	Dexamethasone	Non-COVID-19
Tomazini [10]	2020	brazil	Multicenter	ARDS	CS	20 mg/day, Day1-5; 10 mg/day, Day 6-10 or until ICU discharge	151	85	148	91	Dexamethasone	COVID-19
Steinberg [11]	2006	US	Multicenter	ARDS	CS	2 mg/kg, followed by 0.5 mg/kg, every 6h, for 14 days; 0.5 mg/kg, every 12h, for 7 days	89	26	91	26	Methylprednisolone	Non-COVID-19
Meduri [6]	2007	US	Multicenter	ARDS	Low-dose CS	1 mg/kg/day, up to 28 days Early ARDS (≤ 72 h)	63	15	28	12	Methylprednisolone	Non-COVID-19
Liu [12]	2012	China	Single center	ARDS	CS	100 mg, three times a day, for 7 days	12	2	14	7	Hydrocortisone	Non-COVID-19
Rezk [13]	2013	US	Single center	ARDS	Low-dose CS	1 mg/kg/d, day1-14; 0.5 mg/kg/d, day 15-21; 0.25 mg/kg/d, day 22-25; 0.125 mg/kg/d, day 26-28. Treatment begins in first 48 h	18	0	9	3	Methylprednisolone	Non-COVID-19
Tongyoo [14]	2016	Thailand	Single center	ARDS	Low-dose CS	50 mg, every 6h, for 7 days	98	34	99	40	Hydrocortisone	Non-COVID-19
Meduri [15]	1998	US	Multicenter	ARDS	CS	2 mg/kg/d, day1-14; 1 mg/kg/d, day 15-21; 0.5 mg/kg/d, day 22-28; 0.25 mg/kg/d, day 29-30; 0.125 mg/kg/d, day 31-32.	16	2	8	5	Methylprednisolone	Non-COVID-19
Annane [16]	2006	France	Multicenter	ARDS	Low-dose CS	50 mg hydrocortisone every 6h, for 7 days; And 50 μg of 9-α-fludrocortisone once a day	85	49	92	62	Hydrocortisone and 9-α-fludrocortisone	Non-COVID-19
**Observational study**												
Lamouche-Wilquin [17]	2022	France	Multicenter	ARDS	CS	The mean prednisone-equivalent dosage was 60 ± 48 mg; the median treatment duration was 10 ± 4 days; Treatment begins before or within 24 h after ICU admission	369	76	301	53	Multiple Corticosteroids	COVID-19
Zhang [5]	2022	China	Multicenter	ARDS	Low-dose CS	≤1 mg/kg/day; a median duration of 10 days.	40	11	80	34	Methylprednisolone	Non-COVID-19
Baek [18]	2021	Korea	Single center	ARDS	CS	Methylprednisolone 40 to 180 mg/day or equivalent; A median duration of 13 days (IQR, 6 to 29 days); Treatment began within 7 days of the onset of ARDS	404	177	161	66	Multiple Corticosteroid	Non-COVID-19
Tsai [19]	2020	Thaiwan	Multicenter	ARDS	High-dose CS	≥ 200 mg hydrocortisone equivalent dose within 3 days after ICU admission	85	37	156	30	Multiple Corticosteroid	Non-COVID-19
Hu [20]	2021	China	Multicenter	ARDS	High-dose CS	a cumulative dose ⩾480 mg of methylprednisolone or its equivalent within the first 3 days after ARDS onset.	89	57	175	93	Multiple Corticosteroid	Non-COVID-19
Katz [21]	2022	US	Single center	ARDS	High-dose CS	the equivalent of a dexamethasone dose >10 mg for more than 3 days consecutively.	110	54	95	58	Multiple Corticosteroid	COVID-19
Boglione [22]	2021	Italy	Single center	ARDS	High-dose CS	the dose of 5-8 mg/kg/day of methylprednisolone for 2 consecutive days	31	5	52	12	Methylprednisolone	COVID-19
Varpula [23]	2000	Finland	Single center	ARDS	CS	a dose of 80 mg in the morning and 40 mg in the evening	16	3	15	3	Methylprednisolone	Non-COVID-19
Raurich [24]	2012	Spain	Single center	ARDS	CS	Intravenous methylprednisolone was initiated at a dose of 80 mg in the morning and 40 mg in the evening; Corticosteroid treatment was given 2 mg/kg/day, from day 1-14; 1 mg/kg/d, from day 15-21; 0.5 mg/kg/d, from day 22-28; 0.25 mg/kg/d, from days 29-30; 0.125 mg/kg/d, from days-32.	10	3	9	4	Methylprednisolone	Non-COVID-19
Brun-Buisson [25]	2011	France	Multicenter	ARDS	CS	A median daily dose equivalent to 270 mg hydrocortisone; A median duration of 11 days	83	28	125	21	Multiple Corticosteroid	Non-COVID-19
Takaki [7]	2017	Japan	Single center	ARDS	High-dose CS	1000 mg/day for the first 3 days; From the 4th day, 2 mg/kg/day was administered and gradually tapered off over a period of 1.5 − 2.0 months.	21	14	165	69	Multiple Corticosteroid	Non-COVID-19

* EGC: Experimental group characteristics, TE: Total number of experimental group, EE: Number of events in the experimental group, TC: Total number of control group, EC: Number of events in the control group.

### Pooled results for mortality

**Figure 2.**
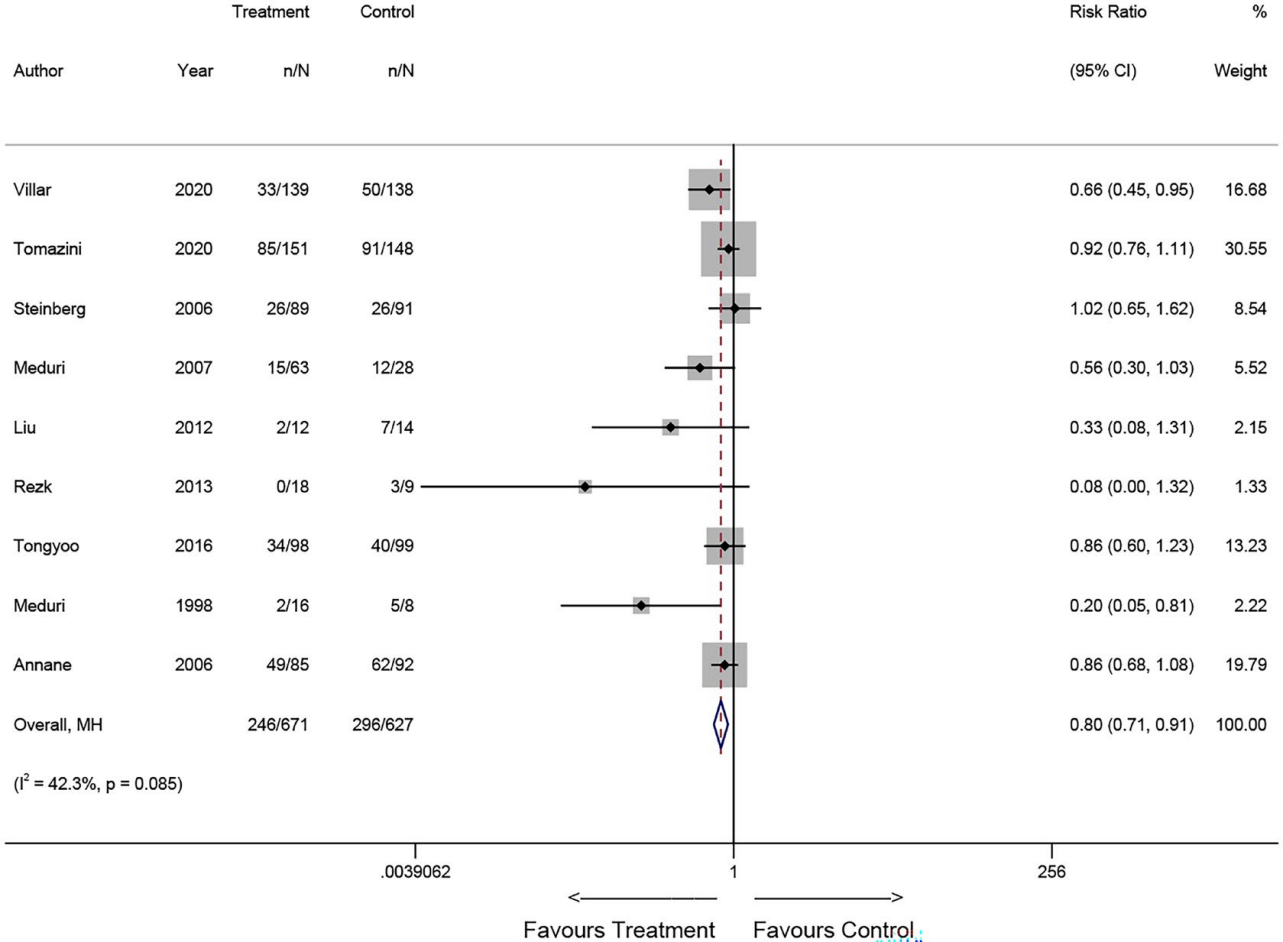
Forest plot illustrating the effects of corticosteroids on mortality of patients with ARDS in RCTs.

**Figure 3.**
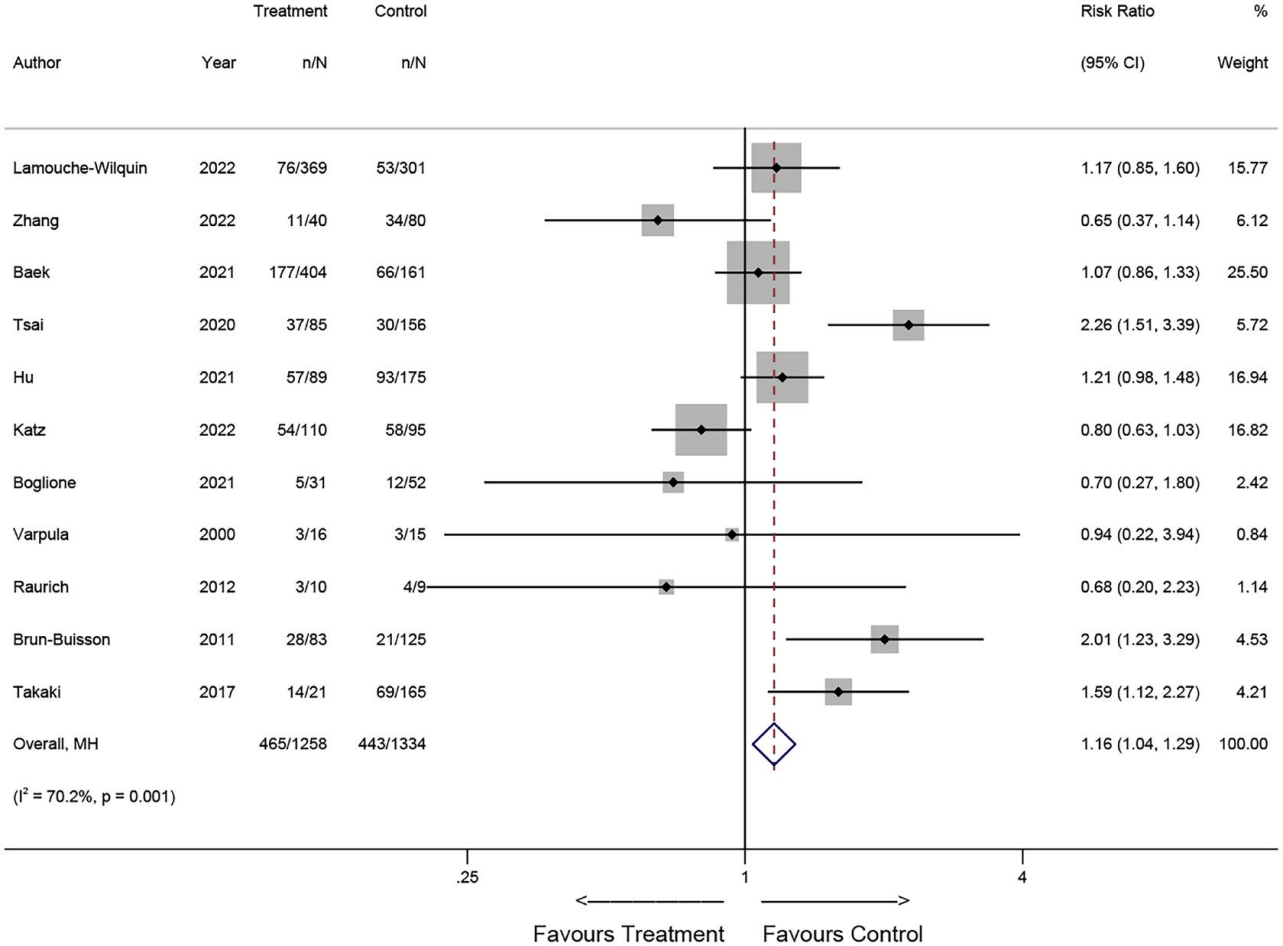
Forest plot illustrating the effects of corticosteroids on mortality of patients with ARDS in observational studies.

Upon pooling the results, an association between corticosteroid use and mortality was observed. Specifically, the findings from RCTs suggested that corticosteroid use may reduce the risk of death (Relative Risk [RR] = 0.80, 95% CI: 0.71-0.91; *p* = 0.001, Figure 2). Conversely, the results of the observational study suggested that corticosteroid use may increase the risk of death in patients (RR = 1.16, 95% CI: 1.04-1.29; *p* = 0.007, Figure 3).

### Subgroup analysis

#### Dosage of corticosteroids

**Figure 4.**
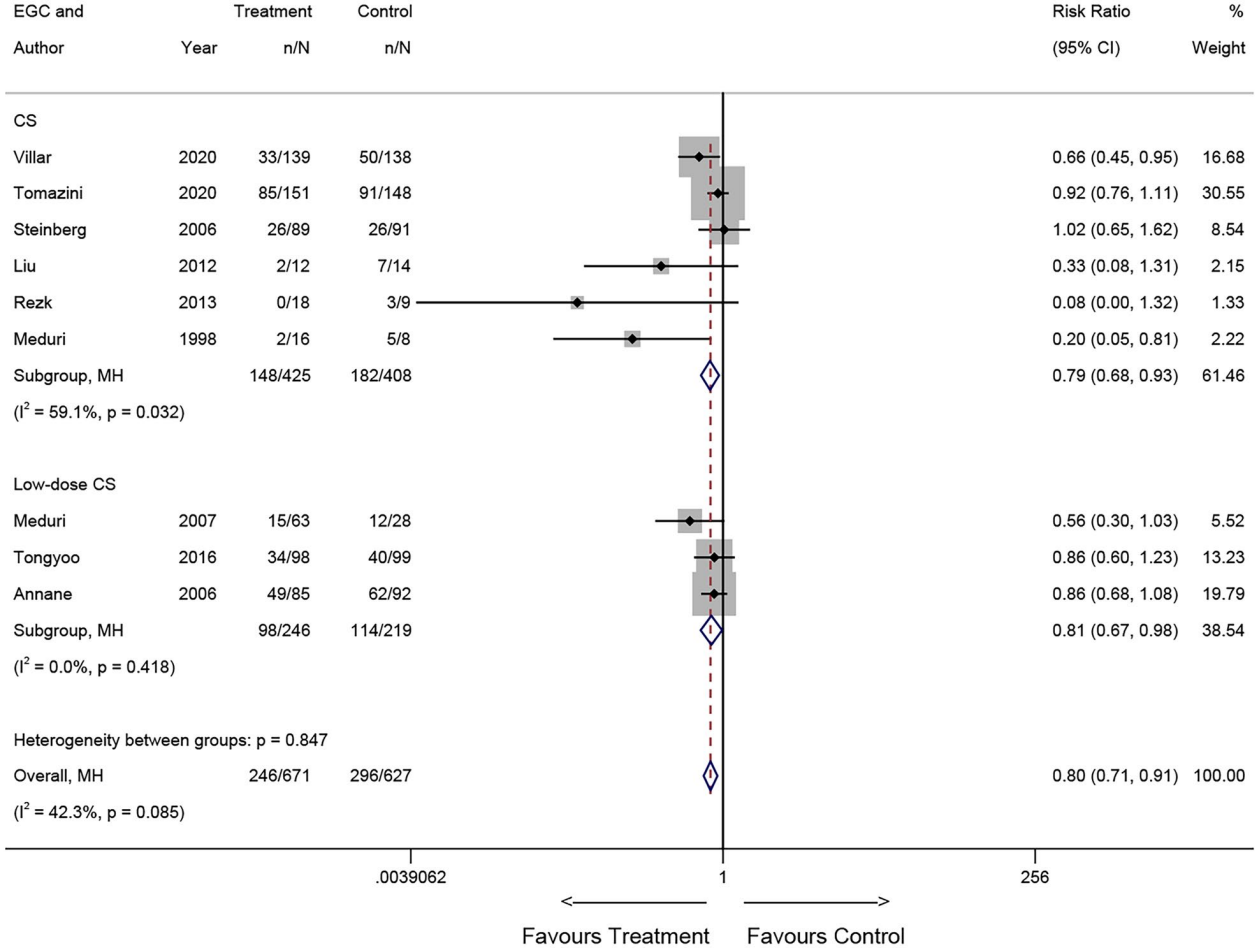
Effects of corticosteroid dosage on mortality in RCTs.

Among the nine RCTs, six studies [5,7,17,19,20,24] included patients who receive regular dosing of corticosteroids, while three studies [6,21,23] included patients receiving low-dose corticosteroids. The meta-analysis revealed a significant association between corticosteroid use and mortality in both subgroups: patients receiving regular corticosteroids (RR = 0.79, 95% CI: 0.68-0.93; *p* = 0.005) and patients receiving low-dose corticosteroids (RR = 0.81, 95% CI: 0.67-0.98, *p* = 0.034). The pooled results indicated that patients in both regular and low-dose corticosteroids subgroups derived benefits from corticosteroid treatment (Figure 4).

**Figure 5.**
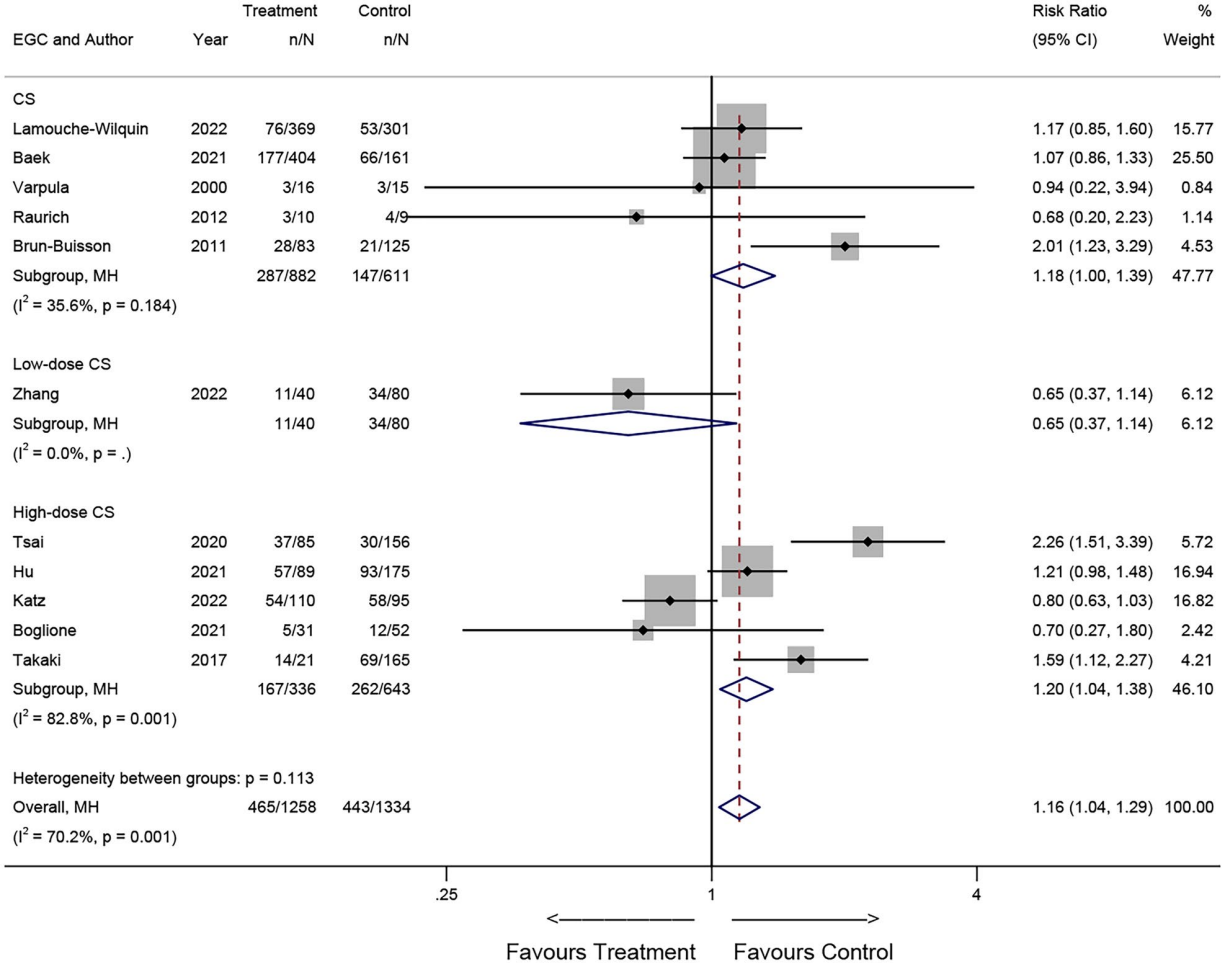
Effects of corticosteroid dosage on mortality in observational studies.

In the 11 observational studies, five studies [10,12–14,22] included patients who receive regular dosing of corticosteroids, five [9,11,15,16,18] receiving high-dose corticosteroids, and one [25] receiving low-dose corticosteroids. The pooled results revealed a significant association between mortality and corticosteroid use in the subgroup of patients on regular dose of corticosteroids (RR = 1.18; 95% CI: 1.00-1.39; *p* = 0.051) and low-dose corticosteroids (RR = 1.20; 95% CI: 1.04-1.38; *p* = 0.010) (Figure 5).

##### Type of corticosteroids

**Figure 6.**
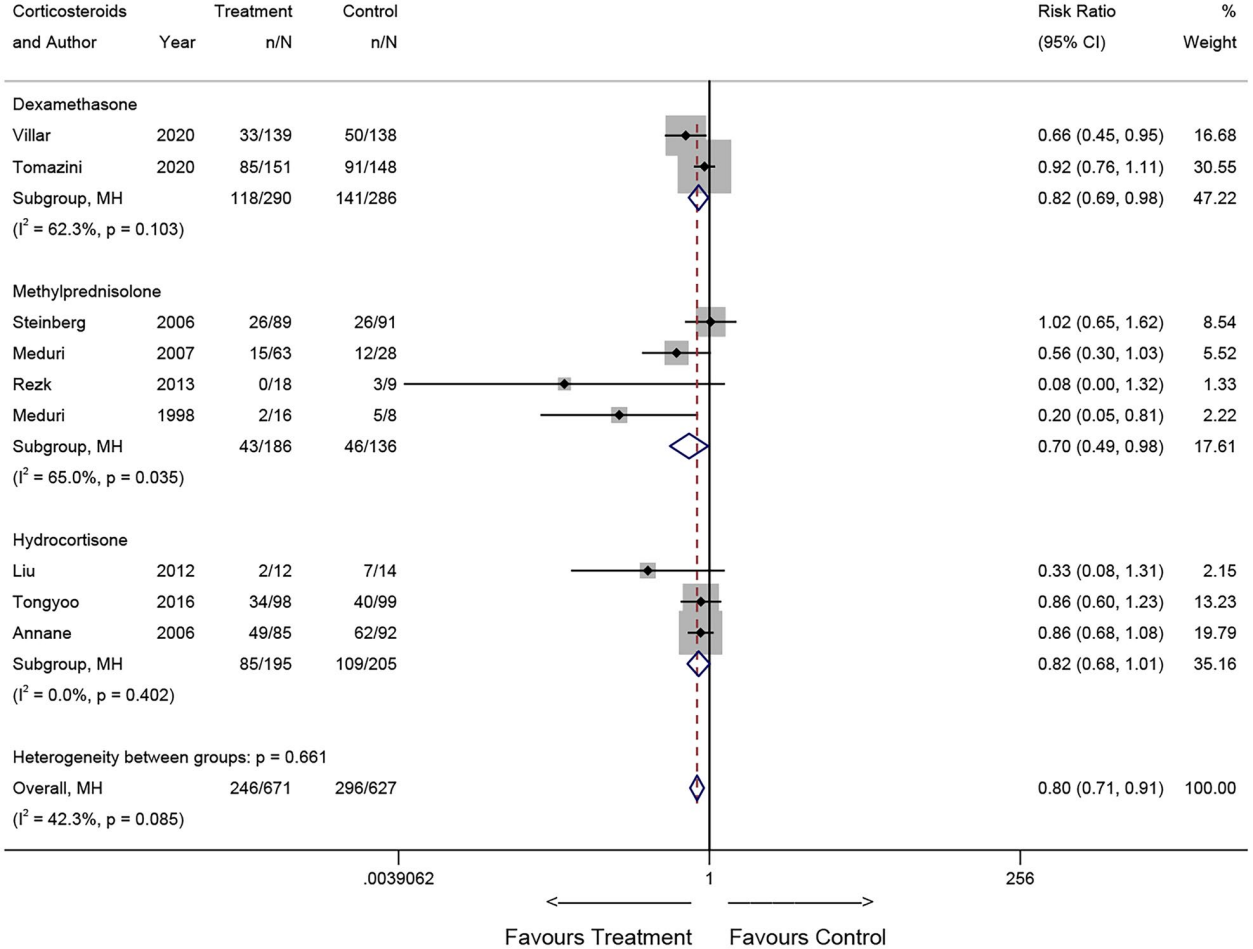
Effects of corticosteroid types on mortality in RCTs.

**Figure 7.**
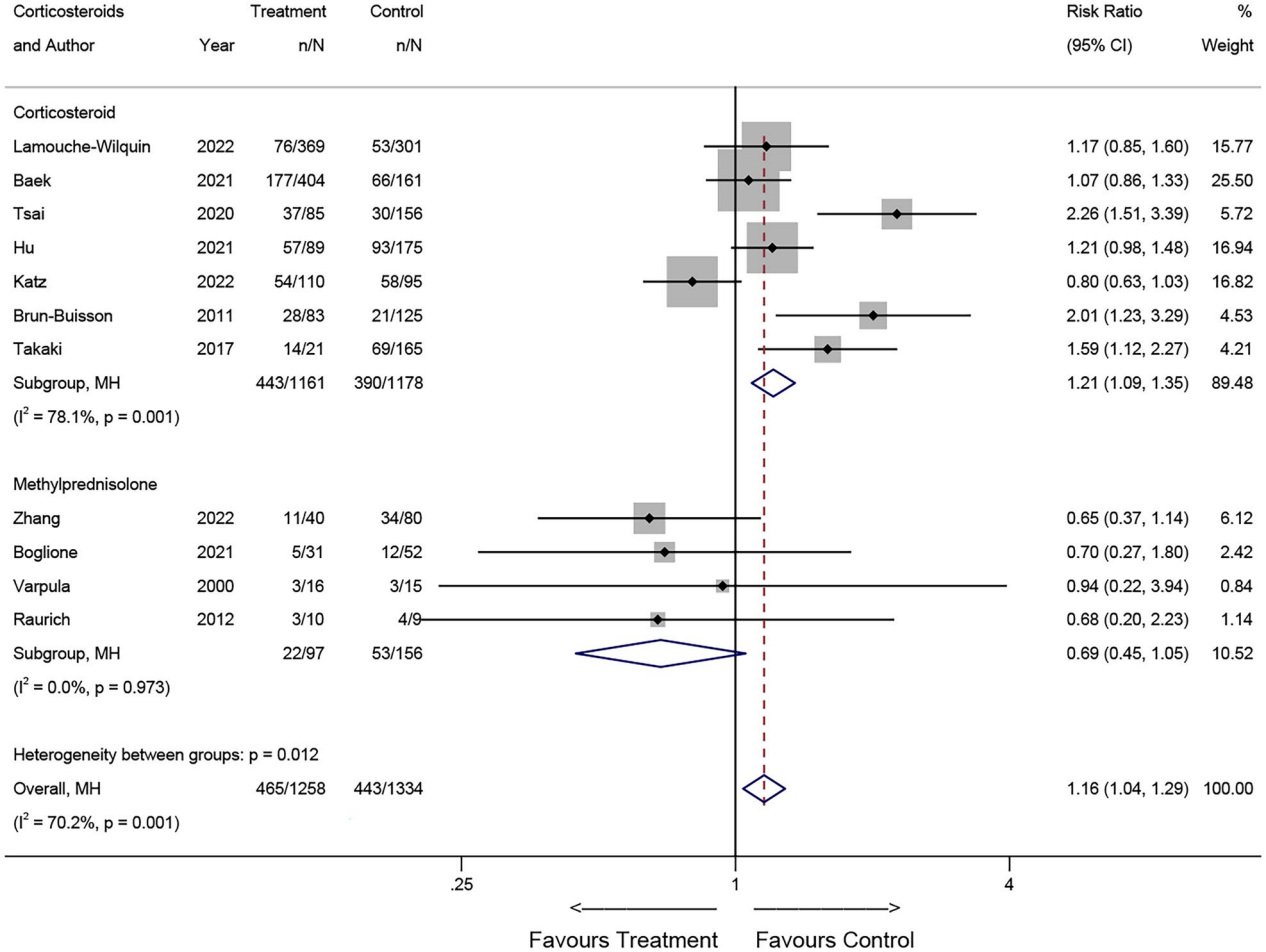
Effects of corticosteroid types on mortality in observational studies.

The results for subgroup analysis regarding the type of corticosteroids are illustrated in Figure 6 for RCTs and Figure 7 for observational studies. In RCTs, the use of dexamethasone and methylprednisolone may reduce the risk of death in patients with ARDS (dexamethasone: RR = 0.82, 95% CI: 0.69-0.98, *p* = 0.029; methylprednisolone: RR = 0.70, 95% CI: 0.49-0.98, *p* = 0.037). For the hydrocortisone subgroup, no association was found. In observational studies, the corticosteroid subgroup (using multiple kinds of corticosteroids) may increase the risk of death (RR = 1.21, 95% CI: 1.09-1.35, *p* = 0.001), whereas no association was found for the methylprednisolone subgroup (RR = 0.69, 95% CI: 0.45-1.05, *p* = 0.084).

#### COVID-19/non-COVID-19

**Figure 8.**
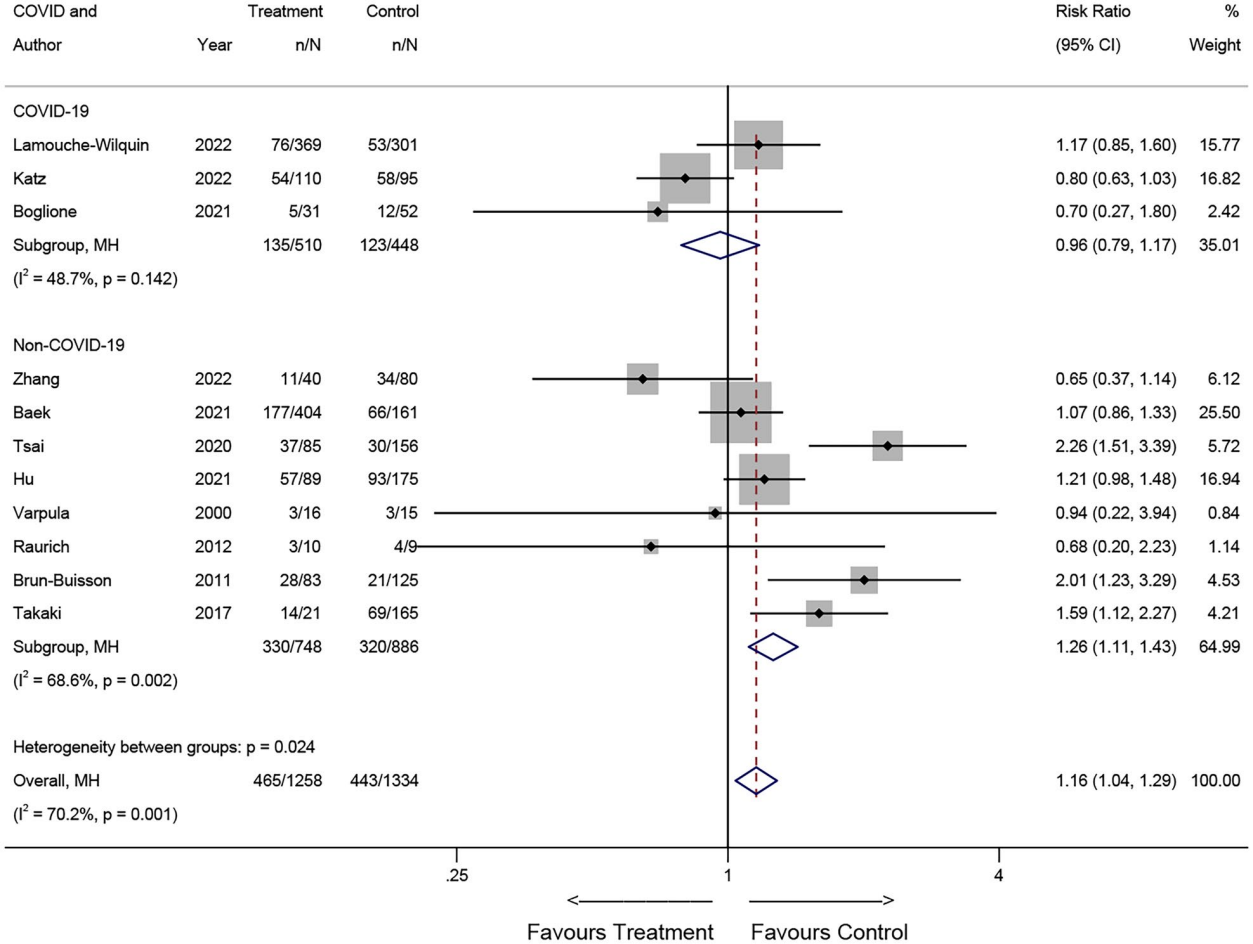
Effects of COVID-19 on mortality in observational studies.

In the observational studies, three studies [10,11,16] included patients with concomitant COVID-19, while eight studies [9,12–15,18,22,25] involved patients without concomitant COVID-19. In the subgroup of patients with COVID-19 corticosteroid treatment did not show significant benefit (RR = 0.96; 95% CI: 0.79-1.17; *p* = 0.700), and there was no substantial heterogeneity observed among these studies (I2 = 48.7%, *p* = 0.142). Conversely, in the subgroup of patients without COVID-19, corticosteroids were associated with an increased risk of death (RR = 1.26; 95% CI: 1.11-1.43; *p* = 0.001), with higher heterogeneity observed (*I^2^* = 68.6%, *p* = 0.002) (Figure 8).

Due to the limited number of studies comprising patients with COVID-19, subgroup analysis regarding COVID-19 was not conducted in RCTs.

### Publication bias

Publication bias was assessed using funnel plot, and no publication bias was found (Figure S1 and S2).

### Risk of bias assessment

We used a modified version of the Cochrane risk of bias tool to assess risk of bias in RCTs, and a revised version of the NOS for observational studies (Table S2). In the nine RCTs, one study had a high risk on blinding of participants and personnel (performance bias) and blinding of outcome assessment (detection bias), two studies had an unclear risk on blinding of participants and personnel (performance bias) and blinding of outcome assessment (detection bias), and all articles had a low risk on the remaining items (Table S3). In the 11 observational studies, two studies scored 0 for non-exposed group selection, two scored 0 for controlling for the major factor, and seven scored 0 for controlling for addition factor. All articles scored 0 for not providing detailed follow-up information.

## Discussion

In this study, we identified and appraised 20 studies involving 3,890 subjects to evaluate the efficacy associated with corticosteroid use in patients with ARDS. The results from RCTs indicated that corticosteroid use was related with a reduction in mortality for patients with ARDS (RR = 0.80, 95% CI: 0.71-0.91, *p* = 0.001). However, in observational studies, corticosteroid was associated with higher patient mortality (RR = 1.16, 95% CI: 1.04-1.29, *p* = 0.001). Furthermore, through subgroup analysis, we identified that the efficacy of corticosteroid treatment was influenced by factors such as the dosage, type of corticosteroids used, and the etiology of the ARDS in the patients. Overall, our study provides important insights into the varied outcomes of corticosteroid use in ARDS patients and emphasizes the need to consider these factors when evaluating the potential benefits and risks of corticosteroid therapy in this patient population.

Different effects of corticosteroid therapy between RCTs and observational studies were observed. In RCTs, the use of corticosteroids may reduce the mortality of patients with ARDS. Several previous meta-analyses [26–29], which also included RCTs, have investigated the impact of corticosteroid therapy on ARDS patient, and their overall findings are consistent with our current study. RCTs are considered the gold standard in clinical research as they minimize bias and confounding factors through random patient allocation into treatment groups. Results from RCTs suggest that in a controlled experimental setting, corticosteroids show promise in improving outcomes for ARDS patients, potentially reducing mortality rates. However, interestingly, in our analysis of observational studies, patients with ARDS did not benefit from the use of corticosteroids in terms of mortality. Prior meta-analyses, such as the one conducted by Ruan et al. [30], which included 10 cohort studies, found that corticosteroids had no effect on ICU mortality (RR = 1.05; 95% CI, 0.74 − 1.49) but non-significantly increased 60-day mortality (RR = 1.30; 95% CI, 0.96 − 1.78). The contrasting findings between RCTs and observational studies highlight the importance of considering the level of evidence when evaluating the efficacy of corticosteroids in ARDS patients. The higher mortality association in the observational studies could be influenced by factors unrelated to corticosteroid treatment. For instance, patients with more severe ARDS might be more likely to receive corticosteroids, which could lead to the observed outcome. Additionally, variations in dosages, treatment durations, or underlying comorbidities may have influenced the results.

In the subgroup analysis of RCTs, the investigation revealed that both methylprednisolone and dexamethasone exhibited the potential to reduce mortality in patients with ARDS, with methylprednisolone displaying superior efficacy. This disparity in effectiveness may be attributed to methylprednisolone’s prolonged *in vivo* residence time and its relatively higher concentration in the pulmonary region [31]. Conversely, hydrocortisone did not exhibit a significant impact. In observational studies, the lack of standardization in treatment protocols introduced variability, leading to inconsistent outcomes. In conclusion, our data suggests that distinct corticosteroid types may yield variable effects on the outcomes of ARDS patients, thereby necessitating individualized corticosteroid selection based on the patient’s specific condition.

Another crucial factor influencing patient outcomes in ARDS is the dosage of corticosteroid therapy, which has undergone changes over recent decades. Pre-1990 studies predominantly employed a high daily dose (30 mg/kg) with a short duration (≤2 days) regimen for ARDS treatment, based on the equivalent doses of methylprednisolone. However, in the subsequent two decades, most studies adopted a protocol involving a daily dose of ≤2 mg/kg, with a gradual taper [30]. In the present study, both low-dose and regular-dose corticosteroid regimens demonstrated protective effects on patients with ARDS. These findings were corroborated by similar results reported by Cui et al. [32].

A noteworthy discrepancy in corticosteroid treatment effects emerged when comparing non-COVID-19 and COVID-19 patients in observational studies. In non-COVID-19 patients, corticosteroids were associated with increased mortality in the experimental group, while no such association was observed in patients with COVID-19. These findings underscore the diverse treatment effects of corticosteroids across different etiologies of ARDS. It is biologically plausible that distinct ARDS etiologies elicit varying responses to corticosteroid therapy [33]. The primary targets of damage differ in ARDS caused by disparate etiologies [34], thus contributing to the differing efficacy of corticosteroid treatment among these etiological subgroups.

### Limitation of this study

Our study has several strengths. Firstly, we implemented a comprehensive search strategy, which ensures a thorough investigation of the existing literature. Secondly, to address potential sources of inconsistency among previous studies and to offer more robust recommendations for the use of corticosteroids in ARDS, we conducted a rigorous subgroup analysis. Thirdly, we included both RCTs and observational studies. This inclusion has served to unveil the disparities between outcomes observed in clinical trials and real-world practice. However, we acknowledge the presence of certain limitations. Notably, the relatively small number of RCTs and the corresponding sample sizes may pose a limitation to the statistical power of our analyses. In certain subgroup analyses, the restricted number of studies, often reduced to only one, warrants cautious interpretation. Secondly, when assessing the etiology-specific response to corticosteroids, the classification of ARDS etiologies was limited due to the diverse mix of study populations across various studies. This could have impacted our ability to fully elucidate the role of corticosteroids in specific ARDS etiologies. Lastly, the quality of included studies might have acted as a confounding factor in our subgroup analysis, which was challenging to control adequately.

## Conclusions

In conclusion, in RCTs, we observed protective effects of corticosteroids on ARDS mortality, suggesting potential benefits in certain subgroups. However, contrasting results were found in observational studies, where an association with increased mortality was evident. These disparate findings between study types underscore the need for caution in routinely administering corticosteroids in ARDS cases. Further research with comprehensive clinical trials is essential to better delineate the most suitable subgroups for corticosteroid therapy in ARDS, ultimately guiding more effective and personalized treatment strategies.

## Supplementary Material

Supplemental Material

## Data Availability

All data generated or analyzed during this study are included in this published article and its supplementary information files.
