# Supplementary material for: Efficacy of corticosteroids in patients with acute respiratory distress syndrome: a meta-analysis
Source: Ann Med. 2024 Aug 21;56(1):2381086. doi: 10.1080/07853890.2024.2381086 (PMC11340212; doi:10.1080/07853890.2024.2381086)

Figure S1. Publication bias analysis for RCTs


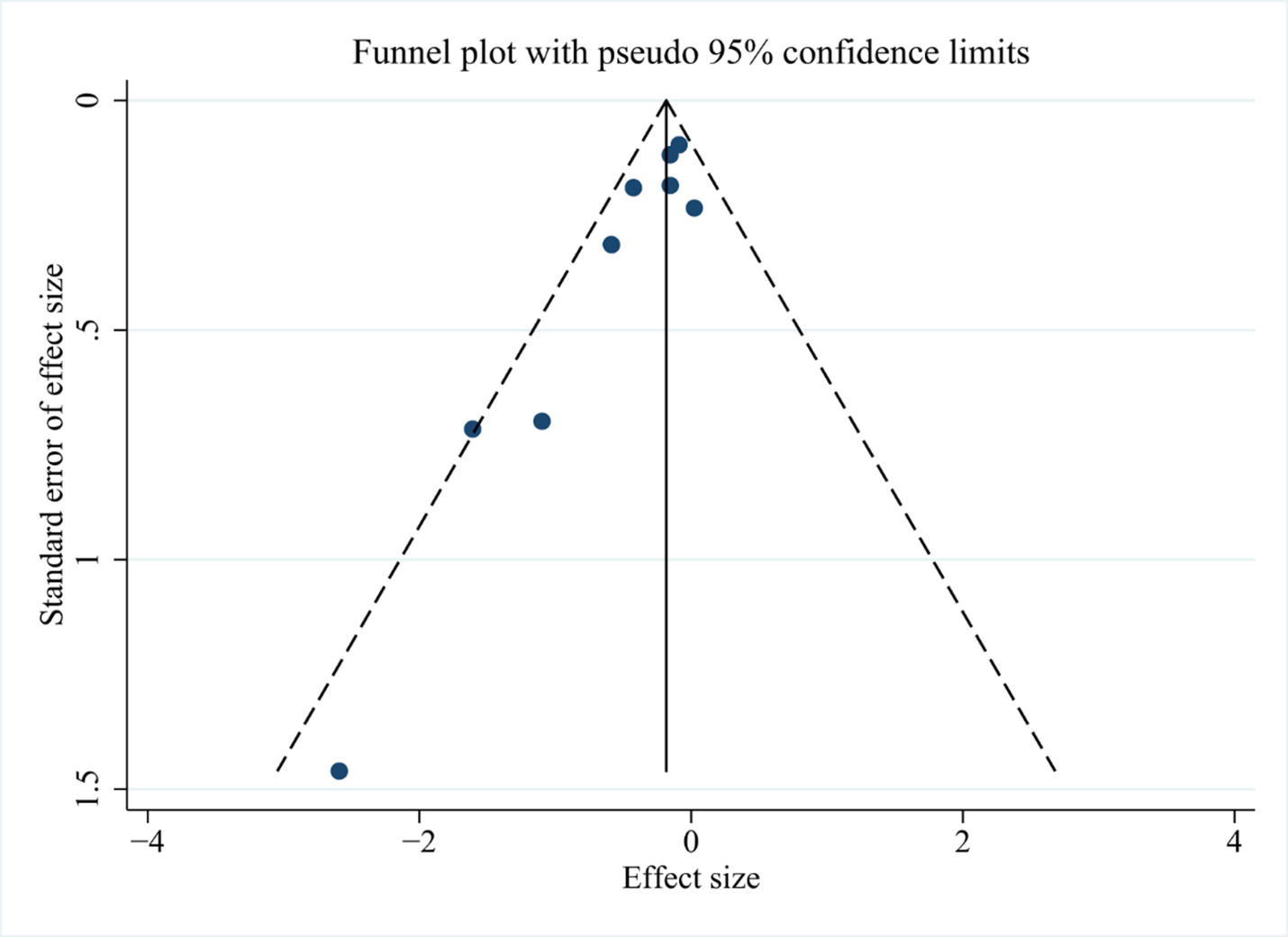


Figure S2. Publication bias analysis for observational studies


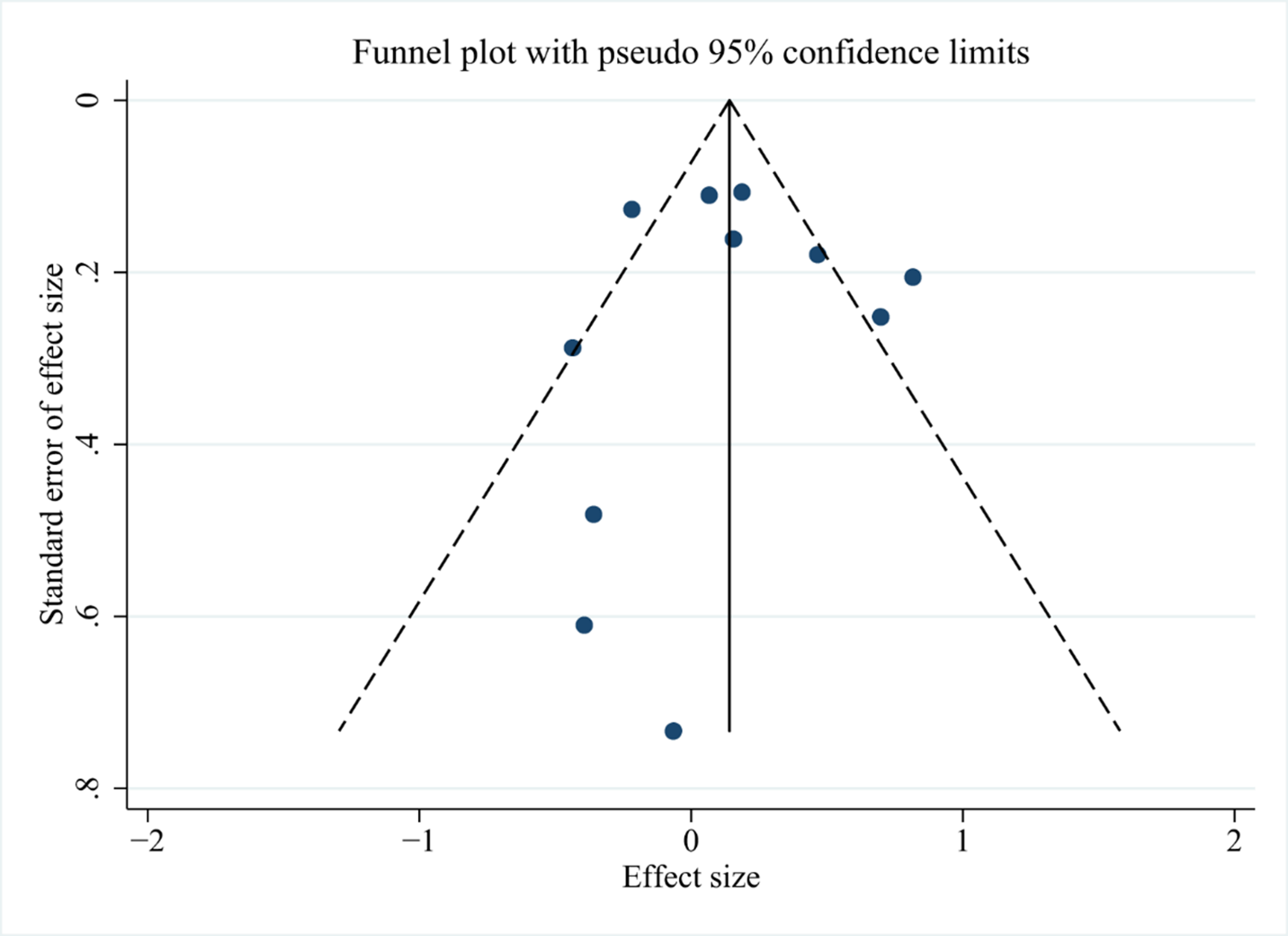

Supplement: Supplemental Material [file IANN_A_2381086_SM8470.zip › suppl_data/Figure S1 and S2.docx]
