# Supplementary material for: Efficacy of corticosteroids in patients with acute respiratory distress syndrome: a meta-analysis
Source: Ann Med. 2024 Aug 21;56(1):2381086. doi: 10.1080/07853890.2024.2381086 (PMC11340212; doi:10.1080/07853890.2024.2381086)
Supplement: Supplemental Material [file IANN_A_2381086_SM8470.zip › suppl_data/Table S1.docx]

**Table S1.** The detailed search strategy used in this study

| **Database** | **Search strategy** |
| --- | --- |
| PubMed | #1 "Adrenal Cortex Hormones"[MeSH Terms] OR "Adrenal Cortex Hormones" OR Cortico* OR hydrocortisone* OR steroid* OR methylprednisolone OR dexamethasone*  #2 "Respiratory Distress Syndrome"[MeSH Terms] OR "Respiratory Distress Syndrome" OR ARDS  #3 "Acute Lung Injury"[MeSH Terms] OR "Acute Lung Injury" OR ALI  #4 #2 OR #3  #5 randomized controlled trial OR clinical trial OR random* OR placebo OR trial OR control* OR group*  #6 #1 AND #4 AND #5 |
| Embase | #1 corticosteroid/exp  #2 Adrenal Cortex Hormone* OR Cortico* OR hydrocortisone* OR steroid* OR methylprednisolone OR dexamethasone*  #3 #1 OR #2  #4 'Acute Lung Injury'/exp  #5 ALI  #6 #4 OR #5  #7 'Respiratory Distress Syndrome'/exp  #8 ARDS  #9 #7 OR #8  #10 randomized controlled trial OR clinical trial OR random* OR placebo OR trial OR control* OR group*  #11 #3 AND #5 AND #9 AND #10  #12 AND 'article'/it  #13 AND 'human'/de |
| Web of Science | TI=(Adrenal Cortex Hormones* OR Cortico* OR hydrocortisone* OR steroid* OR methylprednisolone OR dexamethasone*) AND TS=(Respiratory Distress Syndrome OR ARDS OR Acute Lung Injury OR ALI) AND TS=(randomized controlled trial OR clinical trial OR random* OR placebo OR trial OR control* OR group*) |
