# Supplementary material for: Efficacy of corticosteroids in patients with acute respiratory distress syndrome: a meta-analysis
Source: Ann Med. 2024 Aug 21;56(1):2381086. doi: 10.1080/07853890.2024.2381086 (PMC11340212; doi:10.1080/07853890.2024.2381086)
Supplement: Supplemental Material [file IANN_A_2381086_SM8470.zip › suppl_data/Table S2.docx]

**Table S2.** Results of quality assessment using the Newcastle-Ottawa Scale for observational studies.

| **Study** | **Selection** | | | | **Comparability** | | **Outcome** | | | **Score** |
| --- | --- | --- | --- | --- | --- | --- | --- | --- | --- | --- |
|  | **Exposed** | **Non- Exposed** | **Ascertainment of exposure** | **Start without outcome present** | **Major factor** | **Addition factor** | **Outcome assessment** | **Follow-up length** | **Adequacy of outcome** |  |
| Varpula, 2000 | ★ | ★ | ★ | ★ |  |  | ★ | ★ |  | 6 |
| Raurich, 2012 | ★ |  | ★ | ★ |  |  | ★ | ★ |  | 5 |
| Brun-Buisson, 2011 | ★ | ★ | ★ | ★ | ★ |  | ★ | ★ |  | 7 |
| Takaki, 2017 | ★ |  | ★ | ★ | ★ | ★ | ★ | ★ |  | 7 |
| Lamouche-Wilquin, 2022 | ★ | ★ | ★ | ★ | ★ |  | ★ | ★ |  | 7 |
| Zhang, 2022 | ★ | ★ | ★ | ★ | ★ | ★ | ★ | ★ |  | 8 |
| Baek, 2021 | ★ | ★ | ★ | ★ | ★ |  | ★ | ★ |  | 7 |
| Tsai, 2020 | ★ | ★ | ★ | ★ | ★ | ★ | ★ | ★ |  | 8 |
| Hu, 2021 | ★ | ★ | ★ | ★ | ★ | ★ | ★ | ★ |  | 8 |
| Katz, 2022 | ★ | ★ | ★ | ★ | ★ |  | ★ | ★ |  | 7 |
| Boglione, 2021 | ★ | ★ | ★ | ★ | ★ |  | ★ | ★ |  | 7 |
